# Development of Convenient System for Detecting Yeast Cell Stress, Including That of Amyloid Beta

**DOI:** 10.3390/ijms19072136

**Published:** 2018-07-23

**Authors:** Yen Nhi Luu, Ian Macreadie

**Affiliations:** School of Science, RMIT University, Bundoora, VIC 3083, Australia; s3691025@student.rmit.edu.au

**Keywords:** heat shock response, heat shock protein, Alzheimer’s disease, beta amyloid, yeast

## Abstract

(1) Background: As a model eukaryote, the study of stress responses in yeast can be employed for studying human health and disease, and the effects of various drugs that may impact health. “Reporting” of stress in yeast has frequently utilised enzymes like β-galactosidase that require laborious assays for quantitative results. The use of a stress reporter that can be measured quantitatively and with high sensitivity in living cells in a multi-well plate reader is a more desirable approach; (2) Methods: A multi-copy yeast-*Escherichia coli* shuttle plasmid containing the *HSP42* promoter upstream of the mCherry reporter, along with the *URA3* selectable marker was constructed and tested; (3) Results: Under certain stress conditions inducing the heat shock response, transformants containing the plasmid produced red fluorescence that could be readily quantitated in a microtitre plate reader. Stresses that produced red fluorescence included exposure to heat shock, copper ions, oligomeric amyloid beta (Aβ_42_) and fibrillar Aβ_42_; (4) Conclusions: Being able to conveniently and quantitatively monitor stresses in whole live populations of yeast offers great opportunities to screen compounds and conditions that cause stress, as well as conditions that alleviate stress. While freshly prepared oligomeric amyloid beta has previously been shown to exhibit high toxicity, fibrils have been generally considered to be non-toxic or of low toxicity. In this study, fibrillar amyloid beta has also been shown to induce stress.

## 1. Introduction

Heat shock proteins (HSPs) are ubiquitously expressed and conserved in both yeast and humans [1]. Low level, constitutive expression of HSPs perform housekeeping functions, assisting in maintenance of proteostasis [2]. HSPs target up to 3% of the total number of genes in yeast, with some acting as molecular chaperones to assist in binding of and folding of proteins and sequester misfolded polypeptides towards proteolytic pathways, while others are involved in intracellular transport, cell wall maintenance, and oxidative stress mechanisms [3,4,5]. While HSPs are always present within cells, their expression may be upregulated during the heat shock response (HSR) in response to cellular stress which may include changes in environment, such as elevated temperature [6,7], misfolding or aggregation of proteins [2,3], and reactive oxygen species (ROS) production [8,9]. The HSR is mediated by activity of heat response factors (HRFs) that bind to a 5 bp heat shock element (HSE) in the promotor regions of heat shock genes to initiate transcription [4,10,11].

As a defense mechanism against misfolded and aggregated proteins, HSPs are vital in the response against the toxic Alzheimer’s disease (AD) protein Aβ. Aβ is present in many forms in an AD-affected brain including monomers, toxic oligomeric intermediates and fibrils. The soluble oligomeric form, particularly Aβ_42_, produces cytotoxic effects that initiate a cascade of events that contribute to the development of AD due to a higher propensity to aggregate [12,13,14]. HSPs are found at elevated levels in AD-affected brains, activating microglial phagocytosis and degradation, inhibiting Aβ formation, and slowing down or inhibiting the rate of aggregation, thereby contributing to the clearance of Aβ [3,5,15,16].

β-galactosidase reporter assays have previously been used for measurement of the HSR towards Aβ, in which a yeast HSE was placed upstream of the *lacZ* gene and β-galactosidase levels were measured with and without the presence of Aβ [17]. The use of such an assay is somewhat inconvenient in that a preparation of cell lysate is required as well as several commercial reagents.

The aim of this study was to develop an alternative reporter assay for quick screening of the HSR using the mCherry fluorescence reporter, to measure cell stress in whole living cell populations without a need for any reagent addition. Development of this expression system could allow for quick, high throughput screening assays to determine conditions that may cause stress to cells. The demonstration of the use of this system is outlined as follows.

## 2. Results

### 2.1. Construction of the pYHSRed1 Plasmid

**Figure 1:**
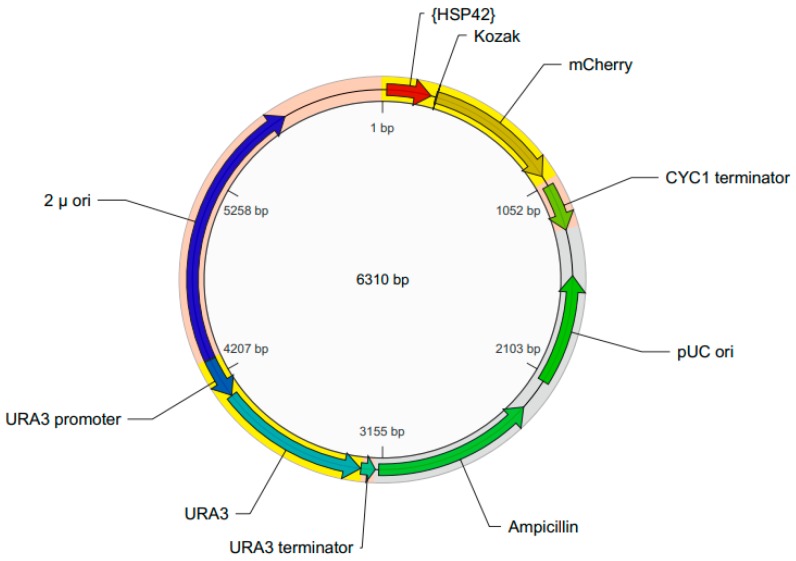
Schematic map of the pYHSRed1 plasmid.

The schematic map of the pYHSRed1 plasmid is shown in Figure 1. It has a 2 µ *ori* for high copy replication in *S. cerevisiae*. The mCherry reporter is located downstream from the promotor of *HSP42*, the most abundant cytosolic HSP in yeast for suppression of aggregation [18]. It contains a *URA3* gene for selection in *S. cerevisiae* strains that have a *URA3* gene mutation or disruption and therefore require uracil supplementation. For propagation of the plasmid in *E. coli* it contains the pUC *ori* and an ampicillin resistance selection marker (encoding β-lactamase). Transformation of this plasmid into a *ura3* mutant *S. cerevisiae* BY4743 strain that requires uracil for growth produces a transformant that no longer requires uracil supplementation. The expression of mCherry expression and red fluorescence should be regulated by the *HSP42* promoter, so the intensity of red fluorescence should indicate the amount of the stress response in the recombinant yeast.

### 2.2. Transformation of Yeast with pYHSRed1 and Basal Expression of mCherry

**Figure 2:**
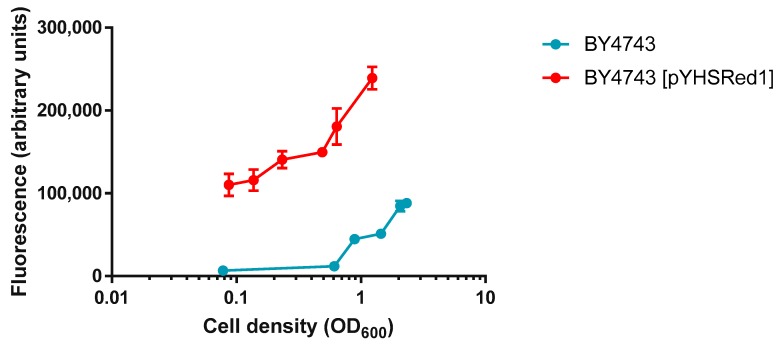
Comparison of basal levels of red fluorescence in BY4743 and BY4743 [pYHSRed1] in cultures of varying cell densities. Mean ±SEM of Data are shown as triplicate measurements.

To examine the basal levels of red fluorescence afforded by pYHSRed1, a comparison was made between BY4743 and its transformant, BY4743 [pYHSRed1]. BY4743 and BY4743 [pYHSRed1] were grown to exponential phase in liquid minimal media with the appropriate supplementation required by each strain, incubated at 30 °C for two hours. Transformants had some production of mCherry, as indicated by the increased red fluorescence (Figure 2). This basal expression of mCherry fluorescence in BY4743 [pYHSRed1] was significantly higher (*p* < 0.05 for all comparisons at same cell density) than that of the untransformed parental BY4743 strain, indicative of the low level constitutive expression of HSPs in yeast cells when unstressed (Figure 2). The measurement of mCherry fluorescence in cultures of varying cell densities was also analysed to determine how the density of cell cultures affected fluorescence. Fluorescence was proportional to cell density, with cell cultures of larger OD_600_ readings producing higher mCherry fluorescence. Arbitrary measurements of higher mCherry fluorescence of the parental BY4743 strain were also observed at greater cell densities which may be attributed to high sensitivity of the spectrophotometer. For measurement of fluorescence in successive experiments, cell cultures of OD_600_ ≥ 0.6 were utilised.

### 2.3. Increased mCherry Fluorescence in Cells Exposed to Heat Shock and Copper Sulphate

**Figure 3:**
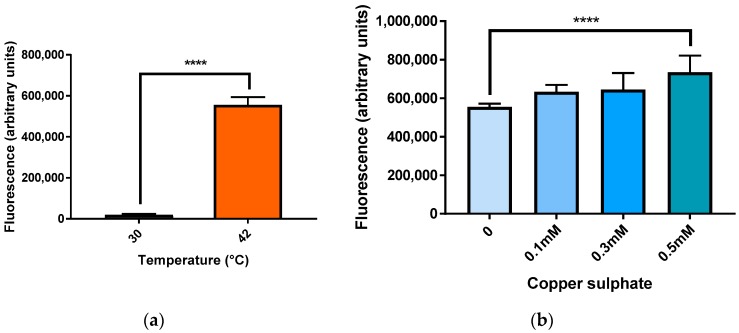
mCherry fluorescence in BY4743 [pYHSRed1] under heat and copper stress. (**a**) Heat stress; (**b**) Stress due to copper sulphate. Data shown as mean ± SEM of triplicate measurements; **** *p* < 0.0001.

The mCherry reporter was examined under conditions that induce HSR in cells: elevated temperatures and exposure to metal ions. BY4743 [pYHSRed1] cells were incubated at 42 °C for two hours, with control cells being incubated at 30 °C. Exposure to 42 °C resulted in a significant increase in mCherry fluorescence being measured, indicating a significant upregulation in heat shock response genes compared to the control (Figure 3a).

Yeast cells were also exposed to copper sulphate for two hours. Treatment with 0.1 and 0.3 mM CuSO_4_ did not produce a significant increase in HSR but cells treated with 0.5 mM CuSO_4_ produced a significant increase in mCherry fluorescence (Figure 3b).

### 2.4. Stress Induced by Oligomeric and Fibrillar Aβ_42_ Measured by mCherry Fluorescence

Elevated mCherry levels were measured when yeast cells were treated with both oligomeric and fibrillar Aβ_42_. Cell responses of both exponential and stationary phase cells were measured due to the differing vulnerability of yeast cells in different growth phases to Aβ_42_ toxicity [19].

**Figure 4:**
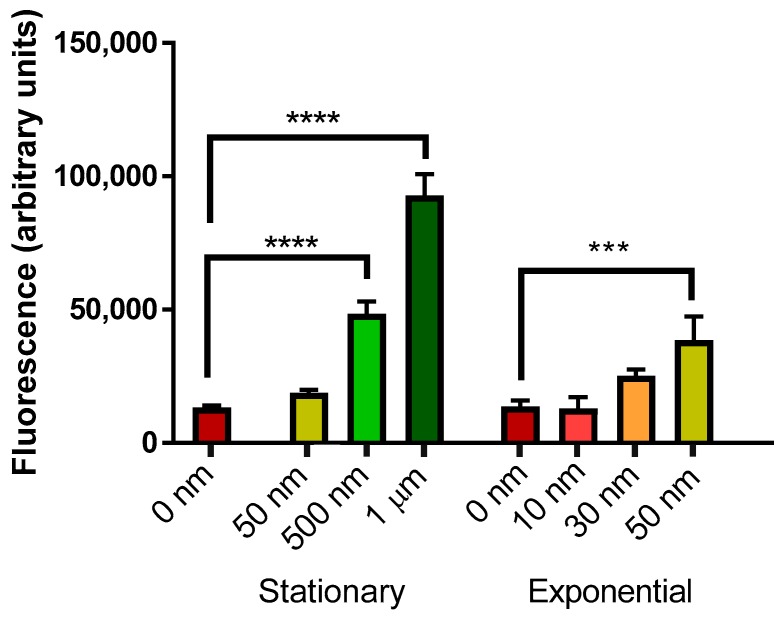
Measurement of mCherry fluorescence of BY4743 [pYHSRed1] cells in stationary and exponential phase growth treated with oligomeric Aβ_42_. Data are shown as mean ± SEM of triplicate measurements; *** *p* > 0.001, **** *p* < 0.0001.

Oligomeric Aβ_42_ (Figure 4) induced a dose-dependent response in mCherry fluorescence in both stationary and exponential phase cells. A significant increase in mCherry fluorescence was observed in stationary phase yeast cells at 500 nM and 1 µM Aβ_42_, but not with 50 nM Aβ_42_. Aβ_42_ also induced significant mCherry fluorescence at 50 nM in exponential phase yeast cells, but there was no significant effect at lower levels.

**Figure 5:**
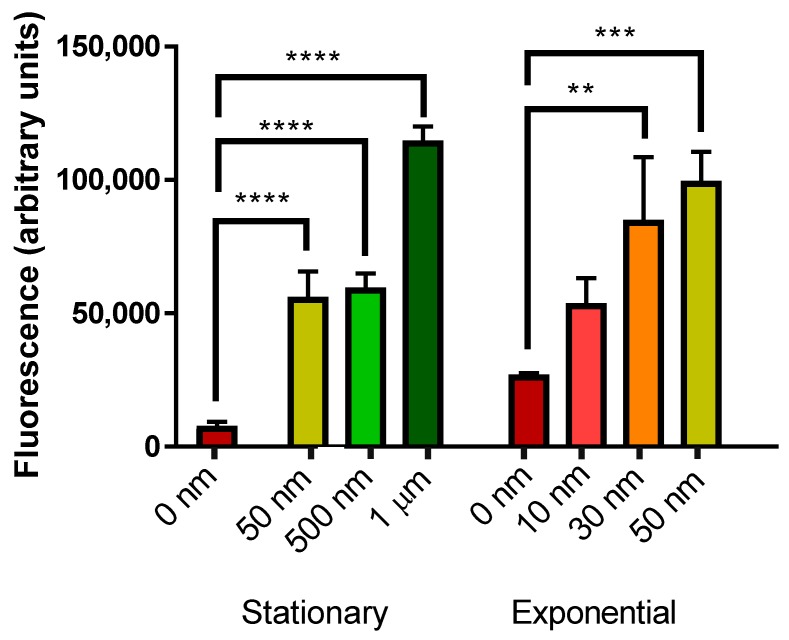
Measurement of mCherry fluorescence of BY4743 [pYHSRed1] cells in stationary and exponential phase growth treated with fibrillar Aβ_42_. Data are shown as mean ± SEM of triplicate measurements; ** *p* > 0.01, *** *p* > 0.001, **** *p* < 0.0001.

Fibrillar Aβ_42_ (Figure 5) induced a significant elevation in mCherry fluorescence in stationary phase yeast cells at 50, 500 nM and 1 µM Aβ_42_. Levels of 30 and 50 nM Aβ_42_ also induced significant mCherry fluorescence in exponential phase yeast cells.

## 3. Discussion

This study aimed to develop a convenient yeast reporter system to measure cell stress correlated with induction of the heat shock response by measuring fluorescence of the mCherry reporter, induced from the HSE of *HSP42*.

Significant basal mCherry expression of the transformant compared to the wildtype was observed, confirming functionality of pYHSRed1 in transformed yeast. Basal expression of HSPs is expected, as in unstressed conditions, HSPs perform housekeeping functions for proteostasis and regulation of protein quality control [9,20].

mCherry fluorescence in BY4743 [pYHSRed1] transformants after exposure to some known inducers of the heat shock response was measured and significant increases in red fluorescence were observed. For example, there was a significant increase in HSR at 42 °C, as this temperature is a heat shock condition in both mammalian and yeast cells and known to activate HSFs [4,21]. Likewise the HSR is also induced by heavy metal ions and oxidants [22], and at 0.5 mM levels it induced highly significant red mCherry expression. Copper can cause stress as it is a heavy metal and may promote oxidative damage at elevated levels in cells [23].

Oligomeric Aβ_42_ is unstable and toxic, with many studies showing its effect in killing of both yeast cells and neurons [24,25,26]. Effects of externally supplemented Aβ_42_ cells may differ based on growth stages, as non-quiescent cells are more susceptible to Aβ_42_ toxicity compared to quiescent cells in the first 24 h of exposure [19]. There is reduced viability of cells exposed to Aβ_42_ oligomers compared to fibrils [24], therefore, lower concentrations of oligomers were applied for treatment. The lower levels of mCherry fluorescence observed compared to fibrillar Aβ_42_ treatment may be due to this cell killing, reducing the number of cells able to emit fluorescence.

In contrast to oligomers, fibrils are generally viewed as harmless and benign, contributing to the non-toxic plaques found in the brain [14]. Though HSPs can sequester oligomer aggregates, they do not cause significant changes to fibrillar Aβ, possibly accumulating on the fibrils due to their inability to process them [3,27]. However, insoluble fibrils may induce oxidative stress from fibrillization [28,29]. Ladiwala et al. [30] also found fibrils formed at elevated concentrations of Aβ were toxic. Fibrils prepared with both HFIP and NH_4_OH pretreatment caused toxicity to *S. cerevisiae* [19]. It is possible that, while not cytotoxic like oligomers, the fibrils cause stress and HSR induction in cells through production of ROS and mild cell killing.

Further work needs to be performed to gain greater understanding of this new attribute of fibrillar Aβ_42_.

## 4. Materials and Methods

### 4.1. pYHSRed1, Yeast Strain and Transformation

The pYHSRed1 was custom designed and produced by VectorBuilder (Cyagen, Santa Clara, CA, USA). It utilises a *URA3* multi-copy VectorBuilder plasmid with 238 nt of the *HSP42* promoter sequences inserted immediately upstream of the mCherry reporter.

The *Saccharomyces cerevisiae* yeast strain BY4743 (*MATa/α his3Δ1/his3Δ1*, *leu2Δ0/leu2Δ0 LYS2/lys2Δ0 met15Δ0/MET15 ura3Δ0/ura3Δ0*) was the host strain used in this study. The plasmid pYHSRed1 was transformed into the host strain as described by Porzoor and Macreadie [31].

### 4.2. Yeast Culture Protocol

Minimal media was used for growth of the BY4743 transformants. The media composition is as follows: Yeast nitrogen base without amino acids (0.67%) and dextrose (2%). For solidification of media agar (1.5%) was added. Supplementation of auxotrophic requirements of BY4743 [pYHSRed1] was performed by adding 20 mg/L histidine and 30 mg/L leucine.

Overnight fresh cultures of the transformants were obtained by inoculating one colony into 10 mL fresh selective minimal media in a 50 mL tube. The tubes were incubated at 30 °C at 250 rpm. Overnight stationary cultures were further grown to exponential phase by transferring 100 µL aliquot to fresh selective minimal media in 15 mL tubes and incubating at 30 °C at 250 rpm for a further two hours.

### 4.3. Preparation of Aβ_42_

Aβ_42_ was pretreated with NH_4_OH as described by [19]. To obtain oligomers, Aβ_42_ was solubilized in water and used immediately. To obtain fibrils, the Aβ_42_ was solubilised in water and incubated at 37 °C for 24 h.

### 4.4. Exposure of Yeast Cells to Heat Shock

Yeast cells were analysed for the effect of exposure to heat shock conditions on mCherry levels. Yeast cells from exponential phase cultures were aliquoted into wells in 96-well microtiter plates. Cells were incubated for a further two hours at 30 and 42 °C.

### 4.5. Exposure of Yeast Cells to Copper Ions

Yeast cells were analysed for the effect of exposure to copper sulphate on mCherry levels. Cells from overnight cultures and exponential phase cultures were suspended in water and then were aliquoted into wells in 96-well microtiter plates. Copper sulphate was added to the diluted cell suspension to required concentrations. The final volume of each well was made up to 200 µL. The microtiter plate was incubated at 30 °C for two hours.

### 4.6. Effect of Exposure to Oligomeric and Fibrillar Aβ on Yeast Cells

Yeast cells were analysed for the effect of exposure to fibrillar and oligomeric Aβ_42_ on mCherry levels. Cells from overnight cultures and exponential phase cultures were pelleted by centrifugation and resuspended in water and aliquoted into wells in 96-well microtiter plates. Oligomeric and fibrillar Aβ_42_ were added to the diluted cell suspension to required concentrations. The final volume of each well was made up to 200 µL. The microtiter plate was incubated at 30 °C for two hours.

### 4.7. Spectrophotometry

Cell density and mCherry fluorescence was measured with the POLARstar omega microplate reader and analysed with BMG Labtech Mars Data Analysis Software (Ortenberg, Germany). Cell density was measured in Corning 96 Well TC-Treated microplates at 600 nm. mCherry fluorescence was measured from a Nunclon Surface black F96 microtiter plate with top optics using an Ex584 excitation filter and 600–680 emission filter.

Raw data was blank corrected, subtracting the mCherry fluorescence reading of the liquid the cell culture was suspended in, to remove background fluorescence. This figure was then divided by the cell density (OD_600_) reading for the culture.

## 5. Conclusions

The heat shock response is vital in both yeast and human cells for defense against various cell stressors, including misfolded and aggregated proteins associated with neurodegenerative diseases such as Alzheimer’s disease. A novel outcome in this study was development of pYHSRed1, a plasmid reporting on stress, especially the HSR, in yeast cells. Coupled with measurement of mCherry fluorescence by a spectrophotometer, the level of stress in live yeast cells may be determined.

Yeast cells containing the pYHSRed1 plasmid were exposed to several conditions known to induce the heat shock response. Elevated temperatures, exposure to metal ions and the subsequent ROS production, and oligomeric and fibrillar Aβ_42_ all induced significant increases in mCherry production, indicative of the upregulation of transcription of heat shock genes. The significant upregulation of mCherry observed after exposure to fibrillar Aβ, considered to be of low or no toxicity, implicates fibrils as a contributor to cellular stress by induction of the HSR.

Transformation of this plasmid into yeast provides an improved method of stress and HSR detection and may be useful for high throughput analysis of therapeutic compounds that may reduce stress caused by Aβ. Future studies could also determine the stress of mutant versions of Aβ on yeast cells and to identify therapeutic compounds that may alleviate the effects of other deleterious proteins, biochemicals or cellular states that cause cellular stress.

## Abbreviations

AD	Alzheimer’s disease
Aβ	Beta-amyloid
HSE	Heat shock element
HSF	Heat shock factor
HSP	Heat shock protein
HSR	Heat shock response
ROS	Reactive oxygen species
